# Plasma Lipidomics Approach in Early and Specific Alzheimer’s Disease Diagnosis

**DOI:** 10.3390/jcm11175030

**Published:** 2022-08-27

**Authors:** Carmen Peña-Bautista, Lourdes Álvarez-Sánchez, Marta Roca, Lorena García-Vallés, Miguel Baquero, Consuelo Cháfer-Pericás

**Affiliations:** 1 Alzheimer’s Disease Research Group, Health Research Institute La Fe, 46026 Valencia, Spain; 2 Division of Neurology, University and Polytechnic Hospital La Fe, 46026 Valencia, Spain; 3 Analytical Unit, Health Research Institute La Fe, 46026 Valencia, Spain

**Keywords:** Alzheimer’s disease, plasma, lipids, diagnosis

## Abstract

Background: The brain is rich in lipid content, so a physiopathological pathway in Alzheimer’s disease (AD) could be related to lipid metabolism impairment. The study of lipid profiles in plasma samples could help in the identification of early AD changes and new potential biomarkers. Methods: An untargeted lipidomic analysis was carried out in plasma samples from preclinical AD (*n* = 11), mild cognitive impairment-AD (MCI-AD) (*n* = 31), and healthy (*n* = 20) participants. Variables were identified by means of two complementary methods, and lipid families’ profiles were studied. Then, a targeted analysis was carried out for some identified lipids. Results: Statistically significant differences were obtained for the diglycerol (DG), lysophosphatidylethanolamine (LPE), lysophosphatidylcholine (LPC), monoglyceride (MG), and sphingomyelin (SM) families as well as for monounsaturated (MUFAs) lipids, among the participant groups. In addition, statistically significant differences in the levels of lipid families (ceramides (Cer), LPE, LPC, MG, and SM) were observed between the preclinical AD and healthy groups, while statistically significant differences in the levels of DG, MG, and PE were observed between the MCI-AD and healthy groups. In addition, 18:1 LPE showed statistically significant differences in the targeted analysis between early AD (preclinical and MCI) and healthy participants. Conclusion: The different plasma lipid profiles could be useful in the early and minimally invasive detection of AD. Among the lipid families, relevant results were obtained from DGs, LPEs, LPCs, MGs, and SMs. Specifically, MGs could be potentially useful in AD detection; while LPEs, LPCs, and SM seem to be more related to the preclinical stage, while DGs are more related to the MCI stage. Specifically, 18:1 LPE showed a potential utility as an AD biomarker.

## 1. Introduction

Alzheimer’s disease (AD) is a complex and multifactorial disease, whose mechanisms of action are currently not fully understood [1]. The most accepted hypotheses describe the accumulation of amyloid-β peptide and phosphorylated Tau (p-Tau) protein in the brain as the cause of the disease [2]. These histological alterations produce neuronal loss, leading to clinical manifestations (memory loss and cognitive decline) [2]. However, when the clinical manifestations are visible, the brain damage is already too great, and current treatments do not show great effectiveness [3]. Currently, the diagnosis of AD is based on cerebrospinal fluid (CSF) biomarkers, neuropsychological evaluations, and neuroimaging [4]. Therefore, there is a need to identify early physiopathological pathways and minimally invasive AD biomarkers.

Lipid metabolism could be related to AD early development since the brain is rich in lipid content, and aging could produce a dysregulation in lipid homeostasis [5]. Therefore, several lipids have been described as potential biomarkers for the disease in different types of biological samples [5]. In fact, the implication of lipids from the cell membrane has been described in APP processing and in amyloid pathology [6]. Several lipid families, such as sphingomyelins (SM), cholesterol esters (CE), phosphatidylcholines (PC), phosphatidylethanolamines (PE), phosphatidylinositols (PI), ceramides (Cer), and triglycerides (TG), have been related to AD [7,8]. These biomarkers could be useful not only for diagnosis but also for disease progression prediction. In fact, LysoPE (LPE) and PE are useful biomarkers for monitoring the conversion of MCI to AD [9], and plasma sphingomyelins have been related to cognitive decline in probable AD patients [10]. In fact, lipidomic analyses have been carried out in order to study the involvement of lipids in AD pathology and progression [11]. Brain tissue from elderly healthy participants and patients with different stages of AD showed differential expression of lipids such as glycerolipids, glycerophospholipids, and sphingolipids [12]. In addition, this research field focusing on these compounds as potential biomarkers in peripheral biofluids (e.g., plasma and serum) is gaining attention [13,14,15].

The aim of this work is to evaluate plasma lipid profiles from untargeted and targeted approaches, identifying lipid families and single lipids involved in early AD as potential biomarkers.

## 2. Material and Methods

### 2.1. Participants and Sample Collection

The participants were between 50 and 80 years old. They were classified into patients with preclinical AD (*n* = 12), patients with mild cognitive impairment (MCI) due to AD (MCI-AD, *n* = 31), and healthy controls (*n* = 20). The clinical assessment consisted of a neuropsychological evaluation based on the Repeatable Battery for Assessment of Neuropsychological Status Delayed Memory (RBANS.DM) [16], Functionality Assessment Questionnaire (FAQ) [17], Mini-Mental State Examination (MMSE) [18], and Clinical Dementia Rating (CDR) [19]. Moreover, NMR-TAC and cerebrospinal fluid (CSF) (β-amyloid-42 peptide, total Tau, and phosphorylated Tau) analyses were carried out. In this sense, patients with preclinical AD show normal cognitive assessments and positive AD biomarkers (CSF and neuroimaging); patients with MCI-AD show impaired cognitive assessments (cutoff for mild cognitive impairment from the scales mentioned above) and positive AD biomarkers; and control participants do not show cognitive impairment and show negative AD biomarkers. Patients with known major neurological or psychiatric conditions were excluded. Informed consent was obtained from all participants, and the Ethics Committee of the Health Research Institute of La Fe (Valencia, Spain) approved the study protocol (2019/0105).

Blood samples were collected from the participants, centrifuged to separate the plasma fractions, and stored at −80 °C until the analysis.

### 2.2. Liquid Chromatography and Mass Spectrometry Analysis

#### 2.2.1. Sample Preparation

The plasma sample treatment was previously described by Peña-Bautista et al. [20]. Briefly, 150 μL of cold isopropanol (IPA) was added to 50 μL of plasma, vortexed, and kept at −20 °C for 30 min. Then, it was centrifuged (13,000× *g*, 10 min, 4 °C), and 90 μL of supernatant was transferred to a 96-well plate. After that, 10 μL of an internal standard (IS) mix solution (17:0 LPC, d18:1/17:0 SM, and 17:0 PE) (100 µg/mL, each compound) was added to each sample. Quality control (QC) was prepared by mixing 10 μL from each plasma sample. A blank was prepared with ultrapure water using the same extraction tube used for blood collection.

#### 2.2.2. Liquid Chromatography

Samples were analyzed by ultra-performance liquid chromatography coupled to time-of-flight mass spectrometry (UPLC-TOF/MS-Orbitrap QExactive Plus MS) following the normalized protocol from the Analytical Unit in Health Research Unit La Fe (Valencia, Spain).

Briefly, the chromatographic conditions consisted of using an Acquity UPLC CSH C18 column (100 × 2.1 mm, 1.7 μm) from Waters. The mobile phase in the positive ionization mode was acetonitrile/water (60:40) with formic acid (10 mM) (A) and isopropyl alcohol/acetonitrile (90:10) with formic acid (10 mM) (B); in the negative ionization mode, it was acetonitrile/water (60:40) with acetic acid (10 mM) (A) and isopropyl alcohol/acetonitrile (90:10) with acetic acid (10 mM) (B). The flow rate was 0.40 mL min^−1^, the column temperature was 65 °C, and the injection volume was 5 µL.

#### 2.2.3. Untargeted Analysis

In the untargeted analysis, the mass spectrometry conditions consisted of positive and negative ionization, an *m/z* range of 70–1700 Da, a resolution full scan of 70,000, a capillary voltage of 2.5 kV, a sheath gas flow rate of 35, an auxiliary gas flow rate of 15, a sweep gas flow rate of 0, a capillary temperature of 250 °C, an s-lens RF level of 65, and an auxiliary gas heater temperature of 200 °C. Samples were randomly injected in the chromatographic system in order to avoid intrabatch variability. Regarding the QC sample, it was analyzed every seven injections to monitor and correct changes in the instrument response. Moreover, it was repeatedly analyzed under the auto MS/MS and all-ion (MSE) fragmentation modes to provide useful information of fragment ions for identification purposes. The stability of the analytical system during the analysis was investigated through the trends and drifts of IS intensities over the course of the batch analysis. A blank analysis was performed at the end of the sequence and was used to identify artefacts from sampling, the preparation of samples, and analysis.

Then, some variables were annotated, with a mass error <5 ppm, and some of them were selected for a subsequent targeted analysis.

#### 2.2.4. Targeted Analysis

Some of previous variables were selected for a targeted analysis through the analysis of chemical standards, attending to the following criteria. First, lipid families that showed statistically significant differences among the participant groups were selected. Then, individual compounds from these families that showed statistically significant differences between groups were selected. In the case of no commercially available standards, similar lipid compounds from the same family were selected.

The sample treatment and the MS/MS method were developed for the simultaneous targeted analysis of seven lipid compounds (18:1 LPE, 18:0 LPC, 16:1 SM (d18:1/16:1), 16:0 SM (d18:1/16:0), 18:0 SM (d18:1/d18:0), 18:1 (9-Cis) PE (DOPE), and 24:0 SM). In addition, 17:0 LPC, 17:0 SM (d18:1/17:0), and 17:0 PE were used as internal standards. Metabolite concentrations were calculated by an internal calibration using a reaction and multiple monitoring (MRM) method. The employed mass spectrometry conditions consisted of positive ionization, a capillary voltage of 3 kV, a sheath gas flow rate of 35, an auxiliary gas flow rate of 15, a sweep gas flow rate, a capillary temperature of 250 °C, an s-lens RF level, and an auxiliary gas heater temperature of 200 °C. The normalized collision energy was 25 for all compounds. The multiple reaction monitoring (MRM) method parameters are summarized in Table 1.

##### Analytical Method Validation

The analytical characteristics assayed during the validation procedure were the linearity range, precision, accuracy, limit of detection (LOD), and limit of quantification (LOQ). The accuracy was evaluated by means of the recovery test. For this, standards were spiked at three concentration levels, and they were analyzed in triplicate. The precision was estimated from the analysis of standards and spiked samples at three concentration levels (i.e., low, medium, and high) in triplicate. The LOD and LOQ were established experimentally as the concentrations required to generate signal-to-noise ratios of 3 and 10, respectively.

### 2.3. Preprocessing and Data Analysis

The results from the untargeted analytical method were converted to the mzXML file format, and the data were processed (peak detection, noise filtering, and peak alignment) using an *in-house* R processing script based in the LipidMS package published by Alcoriza-Balaguer et al. and developed in the Analytical Unit of the Health Research Institute of La Fe (Valencia) [21]. Then, the obtained dataset was filtered, considering the criteria of the coefficient of variation (CV) <30% in the QC samples, the presence of the feature in 60% of the samples in at least one group, and the blank (water processed as a sample). In fact, a fold-change cutoff (biological sample signal/blank signal < 5) was used to remove features that were not sufficiently abundant in the biological samples. After that, a drift correction from QC-based robust locally weighted scatter plot smoothing (LOESS) for data normalization was performed (excluding potential artefacts). Finally, the obtained normalized dataset was annotated and statistically analyzed.

In order to increase the metabolic coverage, two data analysis strategies were used. The variables were identified by two complementary methods in order to identify more metabolites with different polarity ranges. As a first method, annotation using the LipidMS package and statistical analysis was carried out with the variables. As a second method, annotation by means of the variable accurate mass (AM), using the CEU mass mediator database (including the Kegg, LipidMaps, Metlin, and Human Metabolome databases), a mass range of ±5 ppm, and some adducts ([M+H], [M+Na], [2M+NH4], [M+NH4], and [M+H-2O] for the positive ionization mode and [M-H], [M+HCOOH-H], [2M-H], and [M+Na-2H] for the negative ionization mode), was carried out. In this second approach, the identity of the metabolites was confirmed by comparing the obtained MS/MS fragmentation spectra with those predicted and proposed in the databases. In this sense, four annotation confidence levels were evaluated, as proposed by E. Schymanski et al. (2014) [22]. They were level 1 (identified compounds with structures confirmed by AM, MS/MS spectra, retention time (rt), and reference standards); level 2 (compounds putatively annotated through AM and experimental or predicted MS/MS spectra matched with online libraries); level 3 (compounds putatively characterized by AM matched with online databases); and level 4 (unknown compounds) [23,24].

The results from the targeted analytical method were the signal intensities (arbitrary units) obtained for each lipid compound in plasma samples, and their concentrations were determined from the corresponding calibration curves.

### 2.4. Statistical Analysis

Participant’s characteristics (demographic and clinical) were analyzed using the median and interquartile range (IQR) for continuous variables and relative and absolute frequencies for categorical variables. Differences between participant groups (age controls and early AD) were evaluated by means of the Mann–Whitney test for numerical variables and the Chi-square test for categorical variables.

The variables identified by the LipidMS package [21] were grouped into lipid families (CE, Cer, diglycerol (DG), fatty acid (FA), lysophosphatidylethanolamine (LPE), lysophosphatidylcholine (LPC), monoglyceride (MG), PC, PE, PI, SM, and TG). In addition, we calculated the variables monounsaturated (MUFAS), polyunsaturated (PUFAS), and saturated (SFAS) as the sum of levels (MUFAS, PUFAS, and SFAS, respectively), including all previous lipid families. Then, a univariate statistical analysis was carried out for each lipid class (the sum of signals from the individual lipids in each family). Specifically, the Kruskal–Wallis and Mann–Whitney tests were used to compare the lipid levels among the participant groups. From these lipid families, some compounds were selected for the targeted analysis. Similarly, the univariate analysis was based on the Kruskal–Wallis and Mann–Whitney tests for quantitative variables and the Chi-square test for categorical variables. Correlation analyses were carried out by Pearson correlation test. Analyses were carried out with the software IBM^®^ SPSS^®^ Statistics version 20.0 (SPSS, Inc., Chicago, IL, USA). Statistically significant differences were considered from *p* value <0.05 for all analyses.

**Figure 1:**
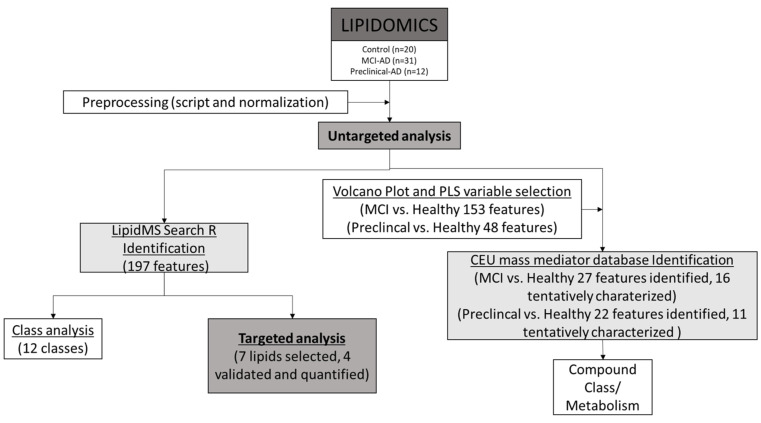
Workflow of the analyses.

On the other hand, a multivariate statistical analysis was carried out with the variables detected in the untargeted analysis in order to identify other potential biomarkers (not identified by the LipidMS package). For this, data from the positive and negative ionization modes were considered simultaneously. First, the normalized variables were visualized in a volcano plot carried out using an *in-house* script in R platform. From this, variables with a stronger combination of fold change (FC) (abs (log2 FC) > 1) and statistical significance (*p* value of *t*-test < 0.05) in each comparison (MCI-AD vs. control and preclinical AD vs. control) were FDR-adjusted and selected for a supervised orthogonal least squares discriminant analysis (OPLS-DA). The OPLS-DA was carried out using Simca 14.1 software (Sartorius Stedim Biotech, Aubagne, France), and it was validated by a seven cross-validation procedure (CV, dataset split into seven subsets). The corresponding models were evaluated by R^2^(Y) (model fit) and Q^2^(Y) (predictive ability) diagnostic indexes, the *p*-value of the CV-ANOVA model, and a permutation test. The most discriminant variables were selected according to their variance importance in projection plot values (VIP > 1.0). Once selected, these features were annotated as potential metabolites by the CEU mass mediator database according to the Schymanski levels of identification [22]. In summary, Figure 1 shows the workflow of these analyses.

## 3. Results

### 3.1. Participant Demographic and Clinical Data

In Table 2, the clinical and demographic characteristics of the participants are summarized. As was expected, neuropsychological variables (CDR, RBANS, FAQ, and MMSE) and CSF biomarkers (amyloid β42, t-Tau, and p-Tau) showed statistically significant differences among the participant groups. In addition, age showed statistically significant differences among the groups. In this sense, the correlations between age and all lipids (from the untargeted and targeted analyses) were assessed, without obtaining significant results for any lipids (see Table S1 in the Supplementary Material).

### 3.2. Lipids Identified by LipidMS Package

**Figure 2:**
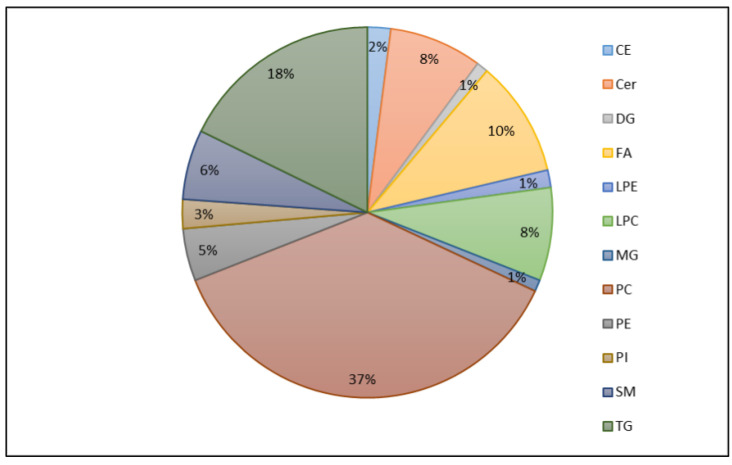
Lipid families identified from untargeted lipidomic analysis and identification by LipidMS package. CE: Cholesterol esters; Cer: Ceramides; DG: Diglycerols; FA: Fatty acids; LPC: Lys phosphatidylcholines; LPE: Lysophosphatidylethanolamines; MG: Monoglycerides; PC: Phosphatidylcholines; PE: Phosphatidylethanolamines; PI: Phosphatidylinositols; SM: Sphingomyelins; TG: Triglycerides.

**Figure 3:**
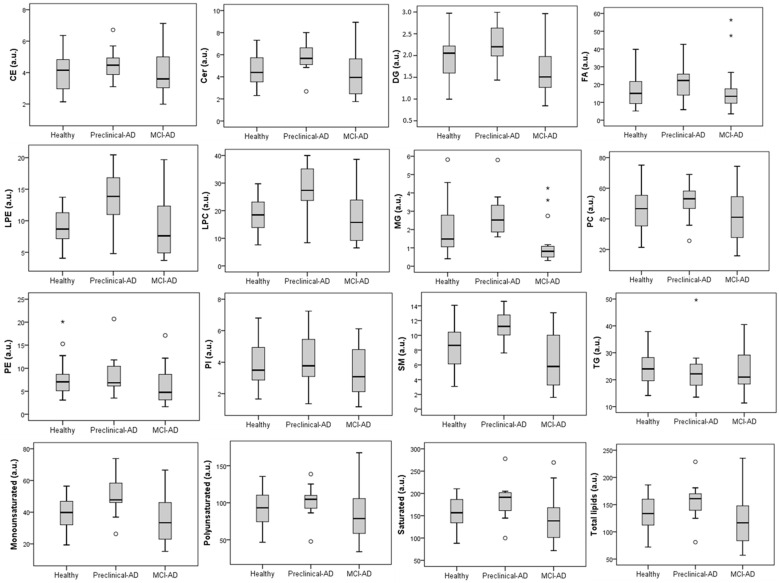
Boxplots representing the levels of lipid families for each participant group (healthy, preclinical AD, and MCI-AD. There were 4 CEs, 4 Cers, 2 DGs, 14 FAs, 3 LPEs, 8 LPCs, 2 MGs, 44 PCs, 7 PEs, 3 PIs, 9 SMs, and 25 TGs included in the analysis (a.u.: arbitrary units)). o: outlayer. *: Extreme outlayer.

From the untargeted analysis, 197 features were annotated by the LipidMS package. They were grouped into some lipid families (4 CE, 16 Cer, 2 DG, 20 FA, 3 LPE, 16 LPC, 2 MG, 73 PC, 9 PE, 5 PI, 12 SM, and 35 TG). As can be seen in Figure 2, the main families were PC (37%), TG (18%), and FA (10%). In Table 3, the DG, LPE, LPC, MG, and SM families and monounsaturated lipids showed statistically significant differences among the three participant groups (preclinical AD, MCI-AD, and healthy). Moreover, the healthy and preclinical AD groups showed statistically significant differences in the levels of the Cer, LPE, LPC, MG, and SM families, while the MCI-AD and healthy groups showed statistically significant differences in the levels of DG, MG, and PE. In addition, Figure 3 shows the boxplots representing the levels of the lipid families in the participant groups (preclinical AD, MCI-AD, and healthy). In general, higher levels were obtained for the preclinical AD group, and lower levels were obtained for the MCI-AD group. A similar tendency was observed for monounsaturated, polyunsaturated, and saturated lipids, although only monounsaturated compounds showed statistically significant differences. In general, a trend was not found for any of the lipid families between the preclinical and MCI groups.

#### 3.2.1. Targeted Analysis

From previous results, the selected lipids were 18:1 LPE, 18:0 LPC, 16:1 SM (d18:1/16:1), 16:0 SM (d18:1/16:0), 18:0 SM (d18:1/d18:0), 18:1 (9-Cis) PE (DOPE), and 24:0 SM. The corresponding analytical method was developed and validated, obtaining satisfactory analytical performance for 18:1 LPE, 18:0 LPC, 16:1 SM (d18:1/16:1), and 16:0 SM (d18:1/16:0) (see Table 4). In fact, the accuracy was satisfactory, with recoveries around 100%, except for 18:0 LPC with recoveries >130%, probably due to the matrix effect. Moreover, a suitable sensitivity was obtained, with LODs between 0.548 and 4.185 nmol L^−1^ and LOQs between 1.83 and 13.95 nmol L^−1^. The other analytes did not show suitable analytical performance (18:0 SM (d18:1/d18:0), 18:1 (9-Cis) PE (DOPE), and 24:0 SM), and they were not determined in plasma samples.

#### 3.2.2. Sample Analysis

A panel of four lipids (previously selected) was determined in plasma samples from healthy participants (n = 20) and patients with preclinical AD (n = 11) and MCI-AD (n = 31). The concentrations of each lipid in the participant groups are summarized in Table 5. As can be seen, statistically significant differences were observed for 18:1 LPE among the three groups (*p* = 0.010) and between the AD (preclinical + MCI) and healthy groups (*p* = 0.003). In addition, this potential AD biomarker showed a correlation with some CSF biomarkers (t-Tau (0.299, *p* = 0.022) and *p*-Tau (0.290, *p* = 0.026)). It should be mentioned that no correlation was observed between the lipids levels and age (see Table S1 in the Supplementary Material).

In addition, LPE 18:1 showed an AUC-ROC of 0.722 (95% CI, 0.595–0.848), discriminating between early AD (preclinical + MCI) and healthy participants.

### 3.3. Compounds Identified by CEU Mass Mediator Database

#### 3.3.1. Preclinical AD vs. Healthy Subjects

**Figure 4:**
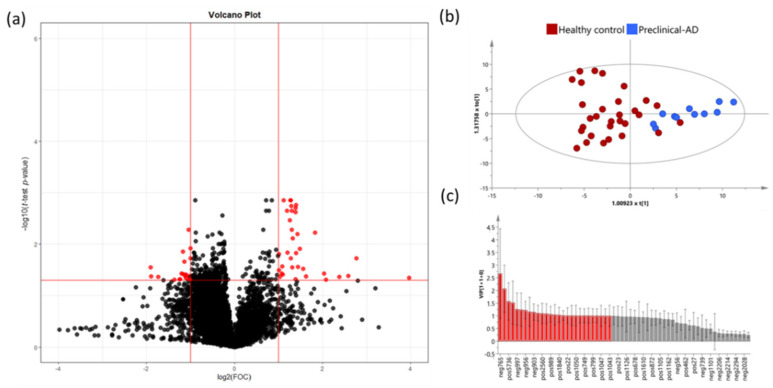
(**a**) Volcano Plot representing the significant variables in the discrimination between healthy controls and preclinical AD participants. Statistically significant variables are represented in red (*p* < 0.05, FC > 2); (**b**) OPLS-DA plot represents differential distribution between healthy controls and preclinical AD; (**c**) Threshold VIP plot value > 1 (red variables).

The volcano plot analysis from the preclinical AD and healthy groups showed 48 significant variables (Figure 4a). The OPLS-DA analysis was carried out with these variables in order to identify the most discriminant variables between the groups. This model showed a *p* value <0.001 and a clear separation between preclinical AD cases and healthy participants (Figure 4b), with good R2Y (0.637) and Q2Y (0.566) parameters. The model was satisfactorily validated (1000 iterations) with R2Y = 0.202 and Q2Y = −0.373.

Potential compounds were subjected to identification and confirmation based on a threshold of VIP value >1 (27 variables) (Figure 4c). Finally, 16 variables were tentatively characterized by querying our experimental MS data with those provided in the commercial databases (see Table S2 in the Supplementary Material). From them, some variables showed more weight over the model (*m*/*z* 1484.140079, 508.3767054, 494.3609278, and 770.6063157). In addition, two variables were putatively annotated through AM and MS/MS mass spectra with online databases. These variables were pisumionoside (*m*/*z* 405.2102471) and 1-O-Palmitoyl-2-O-acetyl-sn-glycero-3-phosphorylcholine (*m*/*z* 520.3404329).

#### 3.3.2. Mild Cognitive Impairment-AD vs. Healthy Controls

**Figure 5:**
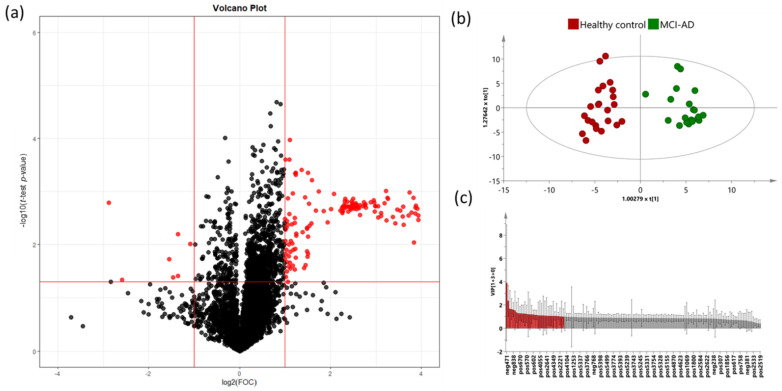
(**a**) Volcano plot representing the significant variables in the discrimination between healthy controls and MCI-AD. Statistically significant variables are represented in red (*p* < 0.05, FC > 2); (**b**) OPLS-DA plot represents differential distribution between healthy controls and MCI-AD. (**c**) Threshold VIP plot value > 1 (red variables).

The volcano plot analysis from the MCI-AD and healthy groups showed 153 significant variables (Figure 5a). The OPLS-DA analysis was carried out with these variables in order to identify the most discriminant lipids between the groups. This model showed a CV *p*-value <0.001 and a clear separation between MCI-AD and healthy control participants (Figure 5b), with good R2Y (0.926) and Q2Y (0.785) parameters. The model was satisfactorily validated (1000 iterations) with R2Y = 0.572 and Q2Y = −0.686.

Potential metabolites were subjected to identification and confirmation based on a threshold of VIP value > 1 (22 variables) (Figure 5c). Finally, 11 variables were tentatively characterized by using the corresponding databases (see Table S3 in the Supplementary Material). From them, some variables showed more weight over the model (*m/z* 409.3113, 362.2550, 350.3417, and 518.351396). In addition, the variable *m/z* 766.573457 was putatively annotated trough AM and MS/MS mass spectra with online databases, and it was identified as a phosphocholine.

## 4. Discussion

A lipidomic approach was developed in plasma samples from participants classified according to their amyloid status (CSF biomarkers) to identify lipid alterations involved in the onset of AD. For this, an untargeted analysis was carried out, and comparisons between early AD (preclinical or MCI) and healthy participants were evaluated. Some significant variables were identified in early AD deregulation, and lipid families were evaluated. Finally, a complementary multivariate analysis was carried out in order to identify other potential discriminative variables.

Lipid families identified by the LipidMS database revealed the potential implication of DG, LPE, LPC, MG, and SM in early AD. In the comparison between preclinical AD and healthy groups, some lipid families were identified as potential biomarkers (Cer, LPEs, LPCs, MGs, and SMs), as they were differentially expressed, especially the monounsaturated species. Similarly, Mielke et al. found an association between Cer and SMs with the risk of AD, although they described differential risks between men and women [25]. In addition, Jazvinšćak Jembrek et al. described the role of ceramides as mediators of neuronal apoptosis related to oxidative stress and Aβ accumulation [26]. Therefore, this deregulation of ceramides in the preclinical stages of the disease could contribute to the advancement of clinical manifestations contributing to neuronal loss. Moreover, Panchal et al. described ceramide accumulation in AD plaques [27]. In addition, SM/ceramide has been related to AD cognitive decline [10]. However, the utility of ceramides as biomarkers for dementias requires further investigation [28]. LPE was described as a biomarker for progression to AD [9], although our results suggest that it could be a potential biomarker for preclinical stages. Similarly, LPCs could be a potential biomarker for the first stages of AD. In this sense, LPCs play a role in polyunsaturated fatty acid (PUFAs) transport across the blood brain barrier, perhaps controlling the availability of these essential compounds for the proper functioning of the brain [29]. In the comparison between MCI-AD and healthy controls, different lipid families were identified as potential biomarkers (DGs, MGs, and PEs). Similarly, Wood et al. found increased levels of DGs and MGs in early AD [30]. PEs could be involved in the physiopathology of AD due to their involvement in cell processes such as oxidative phosphorylation, mitochondrial biogenesis, and autophagy [31]. Our results show that MGs could be potential biomarkers of early AD, including both the preclinical and MCI-AD stages. In addition, LPE, LPC, and SM seem to be more specifically altered in the preclinical stage, while DGs could be useful as biomarkers for the MCI stage.

On the other hand, the annotation of variables by means of other databases (HMDB, Kegg, and Metlin) reported other important annotated variables and metabolite classes. In the discrimination between preclinical AD and healthy subjects, some lipid families were found, such as phosphatidylglicerol, glicerophosphocholine, glicerophosphoserine, phosphoethanolamine, phosphocholine, glicoesphingolipid, diacilglicerol, terpenes, steroids, flavonoid classes, and vitamin E. Specifically, plasma glycerophosphocholine compounds were observed at higher levels in the preclinical AD group. Similarly, other studies showed elevated levels of this lipid in AD brains [32] as well as in cerebrospinal fluid samples from AD patients [33,34], indicating that abnormal phospholipid metabolism in the brain is characteristic of AD. In addition, the present study found that plasma phosphoethanolamine levels were lower in the preclinical AD group, and a previous work found lower levels for PE in AD brain samples [35]. In fact, PE is a precursor for phosphatidylcholine and a substrate for important posttranslational modifications [31]. Moreover, phosphocholine is a precursor of phosphatidylcholine, and higher levels were obtained for the preclinical AD group, indicating a potential membrane impairment in the early disease process [36]. Moreover, glycosphingolipids could be involved in preclinical AD since higher levels were obtained in plasma samples from these participants. In this regard, ceramides, which are involved in sphingolipid metabolism, showed an association with neuropsychiatric symptoms [37]. Moreover, we found higher levels of DGs in the preclinical AD group, similar to the increased plasma levels in early AD, suggesting that lipidomics alterations lead to the accumulation of DGs in MCI subjects [30]. On the other hand, in the present study, phosphatidylglycerol (PG) and flavonoids showed lower plasma levels in the preclinical AD group. Flavonoid compounds could act against AD pathology by inhibiting microglia activation and Aβ aggregation. Therefore, a reduction in these compounds early in the disease may contribute to the development of AD pathways. However, a search of the literature failed to reveal any studies related to this finding. Studies have been reported that vitamin D showed higher levels in preclinical AD compared to healthy participants, but we found that prior investigations reported reduced levels of these vitamins in AD and MIC-AD cases [38]. Since the cases examined here were classified as preclinical AD, it is possible that this group was exhibiting a compensatory response to the disease process. In addition, the discrimination between preclinical AD and healthy controls is characterized by the biomarkers 1-O-Palmitoyl-2-O-acetyl-sn-glycero-3-phosphorylcholine and pisumionoside, which were putatively annotated. Pisumionoside is an exogenous compound derived from vegetables, such as seedpods of garden peas, that could have a hepatoprotective function [39]. These levels are elevated in healthy subjects compared to preclinical AD subjects. Therefore, pisumionoside could have a protective effect against AD. Moreover, 1-O-Palmitoyl-2-O-acetyl-sn-glycero-3-phosphorylcholine is a glycerophosphorylcholine that showed increased levels in AD, in concordance with previous studies [40]. Its oxidized products were considered biomarkers of neuroinflammation in other pathologies such as multiple sclerosis [41]. Moreover, other lipid families (glycosyldiacylglycerols, fatty acids, terpenoids, sesquiterpene mycotoxins, terpene lactones, phosphocholines, glucosylceramides, and fucopentanoses) were annotated by HMDB comparing MCI-AD and healthy groups. First, glycosyldiacylglycerols showed lower levels in the MCI-AD group. Previous studies found an increase in diacylglycerols in the frontal cortex in neurodegenerative diseases such as dementia with Lewy bodies or AD [42]. In addition, glycosylation showed a relationship with neurodegeneration and AD. Therefore, it could be an indicator of disease progression [43]. Moreover, fatty acids showed lower levels in the MCI-AD group, similar to previous reports [44,45], reflecting differences in intake and metabolism. Moreover, terpenoids and some vitamins showed higher levels in the MCI-AD group. In this sense, there is some controversy since previous studies showed protective effects for these compounds [46,47].

Regarding the targeted analysis, the developed analytical method was able to determine low plasma levels of some lipids that could be useful as potential AD biomarkers (18:1 LPE, 18:0 LPC, 16:1 SM (d18:1/16:1), and 16:0 SM (d18:1/16:0)). Accuracy was satisfactory for all of them. However, only 18:1 LPE showed statistically significant increased levels in preclinical and MCI-AD in comparison with healthy controls. Su et al. found this lipid increased in brain-derived extracellular vesicles from AD patients [48]. For LPC in plasma samples, a previous study showed an increase with aging, which is more evident under AD conditions [49]. Similarly, the present study found higher levels of LPC 18:1 and lower levels of L-α-phosphatidilcholine and PC in AD patients. However, Mulder et al. found a decrease in the ratio LysoPC/PC under MCI or dementia due to AD conditions [50]. In addition, the present study showed plasma 18:1 LPC correlations with CSF Tau and p-Tau, which are biomarkers currently employed in AD diagnosis. Specifically, Tau is considered a neurodegeneration biomarker [51]. In this sense, the correlation found between 18:1 LPC and Tau showed the potential utility of 18:1 LPC as a neurodegeneration biomarker. Similarly, previous studies showed the utility of the metabolites 18:0 LPC and 18:2 LPC as potential biomarkers for AD [52]. These discrepancies could be explained by the different types of samples used (plasma and CSF) as well as by the different isomers determined in these compounds’ families. In addition, the ratio between LPC and PC in the plasma samples showed the capacity to differentiate between AD and non-AD participants [53].

The main limitation of this study is the small sample size. However, the participants were accurately classified into groups according to their amyloid status, cognitive state, and brain alterations with neuroimaging. Moreover, there is a lack of confirmation studies to identify the metabolites as reliable AD biomarkers. Nevertheless, this work provides a detailed lipidomic approach from untargeted and targeted analyses that identified potential biomarkers and pathways involved in early AD development. Although analyses of confounding variables, such as age, were not performed, correlations between age and lipids or lipid class were assessed.

## 5. Conclusions

A lipidomic approach was developed from untargeted and targeted analyses of plasma samples. It showed some differential expression of lipids between healthy participants and patients at the early stages of AD. Therefore, the plasma lipid profile could be useful in the early and minimally invasive detection of AD. Among lipid families, relevant results were obtained from DGs, LPEs, LPCs, MGs, and SMs. Specifically, MGs could be potentially useful in AD detection, while LPEs, LPCs, and SM are related more specifically to their preclinical stage and DGs are related to the MCI stage. Among these families, 18:1 LPE showed potential utility as a biomarker for AD and neurodegeneration. In addition, other analyte families, such as phosphatidylglicerol, phosphocholine, glicerophosphocholine, glicerophosphoserine, glicoesphingolipid, vitamin E, terpenes, steroids, flavonoids, glycosyldiacylglycerols, fatty acids, glucosylceramides, and fucopentanoses, showed potential alterations in early AD stages. However, further analysis in a large number of samples is required to validate these preliminary results.

## Figures and Tables

**Table 1:** Acquisition parameters for targeted lipid analysis.

Compound	Mass to Charge (*m/z*) Precursor Ion	Chemical Formula (M)	Product Ion (*m/z*) (Quantitative)	Product Ion (*m/z*) (Qualitative)
18:1 LPE	480.30847	C23H46NO7P	308.294	
18:0 LPC	524.37107	C26H54NO7P	184.073	104.107
16:1 SM (d18:1/16:1)	701.5592	C39H77N2O6P	184.073	104.107
16:0 SM (d18:1/16:0)	703.57485	C39H79N2O6P	184.073	104.107
18:0 SM (d18:1/18:0)	731.60615	C41H83N2O6P	184.073	104.107
18:1 (9-Cis) PE (DOPE)	744.55378	C41H78NO8P	308.294	
24:0 SM	815.70005	C47H95N2O6P	184.073	86.0963
17:0 LPC	568.3626	C25H52NO7P	184.073	
17:0 SM (d18:1/17:0)	717.5905	C40H81N2O6P	184.073	
17:0 PE	720.22537	C39H78NO8P	184.073	

LPE: lysophosphatidylethanolamine; LPC: lysophosphatidylcholine; SM: sphingomyelin; PE: phosphatidylethanolamine; DOPE: dioleoyl phosphatidylethanolamine.

**Table 2:** Clinical and demographic participant characteristics.

		Healthy (*n* = 31)	MCI-AD (*n* = 20)	Preclinical AD (*n* = 11)	*p* Value (Kruskal–Wallis)
Median Age (years) (IQR)		62 (58, 68)	72 (69, 74)	70 (60, 74)	0.000
Gender (Female, *n* (%))		19 (61%)	10 (53%)	6 (50%)	0.737
Educational Level	Primary (*n* (%))	10 (32%)	7 (39%)	4 (33%)	0.023
	Secondary (*n* (%))	7 (23%)	10 (56%)	2 (17%)	
	University (*n* (%))	14 (45%)	2 (18%)	6 (50%)	
Concomitant Medication	Statins (*n* (%))	9 (41%)	12 (63%)	3 (25%)	0.335
	Fibrates (*n* (%))	0 (0%)	3 (17%)	1 (8%)	0.143
	Benzodiazepines (*n* (%))	6 (27%)	3 (16%)	2 (17%)	0.635
	Antidepressants (*n* (%))	7 (32%)	2 (11%)	0 (0%)	0.085
	Antiepileptics (*n* (%))	1 (5%)	0 (0%)	0 (0%)	0.547
	Antihypertensives (*n* (%))	7 (32%)	9 (50%)	2 (29%)	0.424
	Corticoids (*n* (%))	1 (5%)	0 (0%)	0 (0%)	0.547
	Anti-inflammatories (*n* (%))	3 (14%)	0 (0%)	0 (0%)	0.151
Comorbidities	Dyslipidemia (*n* (%))	11 (50%)	11 (58%)	3 (43%)	0.766
	Diabetes (*n* (%))	3 (14%)	2 (11%)	0 (0%)	0.589
	Hypertension (*n* (%))	8 (36%)	9 (47%)	2 (29%)	0.628
	Heart Disease (*n* (%))	1 (5%)	0 (0%)	0 (0%)	0.547
	Cerebrovascular (*n* (%))	1 (5%)	0 (0%)	0 (0%)	0.547
Smoke (Yes, *n* (%))		6 (27%)	3 (16%)	1 (14%)	0.598
Alcohol (Yes, *n* (%))		6 (27%)	2 (11%)	0 (0%)	0.157
Depression (Yes, *n* (%))		5 (23%)	5 (26%)	2 (29%)	0.939
Anxiety (Yes, *n* (%))		4 (18%)	3 (16%)	2 (29%)	0.757
Amyloid β42 (pg mL^−1^) Median (IQR)		1224 (964, 1421)	495 (452, 622)	572 (383, 694)	0.000
t-Tau (pg mL^−1^) Median (IQR)		212 (181, 259)	578 (449, 793)	444 (208, 611)	0.000
p-Tau (pg mL^−1^) Median (IQR)		34 (25, 39)	91 (62, 109)	74 (28, 94)	0.000
CDR Median (IQR)		0.5 (0, 0.5)	0.5 (0.5, 0.5)	0.5 (0, 0.5)	0.001
MMSE Median (IQR)		29 (28, 29)	24 (22, 25)	29 (27, 30)	0.000
RBANS.DM Median (IQR)		98 (94, 103)	42 (40, 53)	95 (87, 101)	0.000
FAQ Median (IQR)		1 (0, 4)	7 (5, 10)	1 (0, 3)	0.000

IQR: Inter-quartile range; AD: Alzheimer Disease; MCI-AD: mils cognitive impairment due to Alzheimer Disease; CDR: Clinical Dementia Rating; MMSE: Mini-Mental State Examination; FAQ: Functionality Assessment Questionnaire; RBANS: Repeatable Battery for Assessment of Neuropsychological Status; DM: Delayed memory.

**Table 3:** Average sum of the different lipid families’ levels in the participant groups (preclinical AD, MCI-AD, and healthy).

Lipid Family	Healthy Controls (HC) (*n* = 31)	MCI-AD (*n* = 20)	Preclinical AD (*n* = 11)	*p* Value (Kruskal–Wallis)	Healthy vs. Preclinical AD (Mann–Whitney, *p* Value)	Healthy vs. MCI-AD (Mann–Whitney, *p* Value)
CE (a.u.)	4.15 (2.86, 4.83)	3.60 (3.03, 5.04)	4.47 (3.86, 4.96)	0.416	0.350	0.685
Cer (a.u.)	4.39 (3.52, 4.39)	3.94 (2.42, 5.75)	5.67 (5.09, 6.87)	0.070	0.038 *	0.452
DG (a.u.)	2.05 (1.56, 2.22)	1.51 (1.25, 1.98)	2.20 (1.94, 2.73)	0.007 *	0.155	0.023 *
FA (a.u.)	15.04 (9.29, 22.21)	13.42 (9.44, 18.38)	22.32 (11.48, 26.24)	0.299	0.201	0.685
LPE (a.u.)	8.68 (7.16, 11.41)	7.61 (4.77, 12.73)	13.86 (10.32, 17.10)	0.006 *	0.002 *	0.418
LPC (a.u.)	18.48 (13.62, 12.39)	15.75 (8.93, 24.98)	27.37 (22.68, 35.24)	0.006 *	0.001 *	0.396
MG (a.u.)	1.48 (1.02, 2.83)	0.81 (0.48, 1.10)	2.52 (1.77, 3.56)	<0.001 *	0.017 *	0.002 *
PC (a.u.)	46.66 (35.34, 56.80)	41.08 (27.78, 55.27)	53.13 (43.75, 59.73)	0.202	0.257	0.316
PE (a.u.)	7.04 (5.09, 8.78)	4.76 (3.05, 9.53)	6.85 (6.13, 10.46)	0.061	0.572	0.034 *
PI (a.u.)	3.50 (2.86, 4.99)	3.08 (2.09, 5.00)	3.77 (2.70, 6.13)	0.366	0.553	0.307
SM (a.u.)	8.63 (6.13, 10.48)	5.79 (3.13, 10.02)	11.21 (9.65, 12.90)	0.001 *	0.003 *	0.061
TG (a.u.)	24.05 (19.40, 28.94)	21.00 (18.36, 29.71)	22.21 (17.83, 27.27)	0.625	0.381	0.537
Monounsaturated (a.u.)	39.78 (31.30, 47.49)	33.35 (22.55, 46.09)	47.79 (45.98, 60.65)	0.011 *	0.009 *	0.232
Polyunsaturated (a.u.)	93.13 (74.29, 113.90)	78.75 (58.62, 106.44)	104.67 (88.91, 111.74)	0.170	0.233	0.307
Saturated (a.u.)	156.73 (132.57, 189.15)	138.36 (99.15, 168.83)	191.35 (155.78, 203.83)	0.100	0.054	0.452

a.u.: arbitrary units. * *p* < 0.05. HC: healthy control.

**Table 4:** Analytical method validation.

Analyte	Standard Concentration (nmol L^−1^)	Recovery (%)	LOD (nmol L^−1^)	LOQ (nmol L^−1^)	Linearity Range (nmol L^−1^)	Equation (y = a + bx) a ± s_a_ b ± s_b_ R^2^
18:1 LPE	6.25	108 ± 14	0.548	1.83	1.83–26.30	0.0019 ± 0.0008
	9.38	109 ± 15				0.0027 ± 0.000063
	12.5	104 ± 17				0.998
18:0 LPC	50	153 ± 15	4.185	13.95	13.95–209.38	0.012 ± 0.024
	75	147 ± 15				0.0072 ± 0.00022
	100	134 ± 21				0.997
16:1 SM (d18:1/16:1)	50	101 ± 11	2.857	9.52	9.52–208.11	0.0774 ± 0.021
	75	101 ± 11				0.0064 ± 0.00019
	100	96 ± 16				0.997
16:0 SM (d18:1/16:0)	12.5	108 ± 58	1.240	4.13	4.13–52.51	−0.0041 ± 0.0063
	18.75	102 ± 6				0.012 ± 0.00024
	25	82 ± 5				0.999
18:0 SM (d18:1/d18:0)	3.13		0.289	0.96	0.96–13.23	0.0014 ± 0.0011
	4.69	100 ± 26				0.0047 ± 0.00017
	6.25	119 ± 59				0.996
18:1 (9-Cis) PE (DOPE)	0.78		0.069	0.23	0.23–3.30	0.00019 ± 0.00015
	1.17	103 ± 65				0.0024 ± 0.000089
	1.56	62 ± 62				0.996
24:0 SM	6.25		0.306	1.02	1.02–26.02	0.24 ± 0.03
	9.38					0.044 ± 0.003
	12.50					0.990

**Table 5:** Lipid concentrations in plasma from participant groups (healthy, MCI-AD, and preclinical AD).

Lipids	Healthy Control (HC) (*n* = 31) Median (IQR) (nmol L^−1^)	MCI-AD (*n* = 20) Median (IQR) (nmol L^−1^)	Preclinical AD (*n* = 11) Median (IQR) (nmol L^−1^)	Kruskal-Wallis *p* Value (Three Groups)	Mann–Whitney *p* Value (AD vs. Non-AD)
18:1 LPE	1.37 (0.38, 1.83)	1.8 (1.2, 4.2)	1.8 (0.9, 3.7)	0.010 *	0.003 *
18:0 LPC	67 (61, 80)	65 (56, 96)	81 (60, 105)	0.504	0.569
16:1 SM	15 (7, 27)	13 (8, 24)	19 (15, 25)	0.501	0.647
16:0 SM	177 (137, 206)	168 (132, 213)	209 (159, 239)	0.374	0.371

* *p* value < 0.05.

## Data Availability

Data are available in the BioStudies public repository with the accession number S-BSST877.

## Supplementary Materials

The following supporting information can be downloaded at: https://www.mdpi.com/article/10.3390/jcm11175030/s1, Table S1: Correlation analysis between age and lipid class or targeted lipids.; Table S2: Metabolites’ annotation from metabolome comparison of preclinical-AD vs. healthy subjects; Table S3: Metabolites’ annotation from metabolome comparison of MCI-AD vs. healthy subjects.

## Abbreviations

AM	Accurate Mass
AD	Alzheimer’s Disease
CDR	Clinical Dementia Rating
CE	Cholesterol Esters
Cer	Ceramides
CSF	Cerebrospinal Fluid
DG	Diglycerols
FA	Fatty Acids
FAQ	Functionality Assessment Questionnaire
IPA	Isopropanol
IQR	Interquartile Range
LOD	Limit of Detection
LOQ	Limit of Quantification
LPC	Lysophosphatidylcholines
LPE	Lysophosphatidylethanolamines
MCI	Mild Cognitive Impairment
MG	Monoglycerides
MMSE	Mini-Mental State Examination
OPLS-DA	Orthogonal Partial Least-Squares Discriminant Analysis
PC	Phosphatidylcholines
PE	Phosphatidylethanolamines
PI	Phosphatidylinositols
QC	Quality Control
RBANS	Repeatable Battery for Assessment of Neuropsychological Status Delayed Memory
SM	Sphingomyelins
TG	Triglycerides
TOF-MS	Time-of-Flight Mass Spectrometry
UPLC	Ultra-Performance Liquid Chromatography
VIP	Variance Importance in Projection
