# Supplementary material for: Plasma Lipidomics Approach in Early and Specific Alzheimer’s Disease Diagnosis
Source: J Clin Med. 2022 Aug 27;11(17):5030. doi: 10.3390/jcm11175030 (PMC9457360; doi:10.3390/jcm11175030)
Supplement: Supplementary file 1 [file jcm-11-05030-s001.zip › jcm-1812401-supplementary.pdf]

Table S1. Correlation analysis between age and lipid class or targeted lipids.

Lipid class/ lipids	Pearson Correlation coefficient (r)	P value
Ces	0.144	0.265
Cer	0.138	0.286
DGs	-0.024	0.851
Fas	0.194	0.132
LPEs	0.117	0.365
LPCs	0.109	0.398
MGs	0.058	0.657
PCs	0.050	0.702
PEs	-0.020	0.876
PIs	-0.060	0.642
SMs	-0,006	0.964
TGs	0.058	0.657
Monounsaturated	0.104	0.421
Polyunsaturated	0.107	0.408
Saturated	0.113	0.383
Total Lipids	0.114	0.377
18:1 LPE	0.245	0.055
18:0 LPC	0.042	0.747
16:1 SM (d18:1/16:1)	0.065	0.616
16:0 SM (d18:1/16:0)	0.023	0.860

Table S2. Metabolites´ annotation from metabolome comparison of preclinical-AD *vs* healthy subjects.

Mass (*m/z*)	RT (min)	Adduct ion	Formula	Identification of variables		Compound class / Metabolism	FC[a]
				Metabolite annotation	Level #		
375.0418	1.61	-	-	NA	4	-	5.998
698.5007	2.28	M+NH4	C35H69O10P	Different PGs	3	Phosphatidylglycerol	2.462
423.1395	0.79	M+Na-2H	C22H26O7	Sphenostylin D<br>Antiarone K	3	Flavonoids	2.625
795.3354	0.80	2M+Na	C22H26O6	3,5-Di-O-methyl-8-prenylafzelechin-4beta-ol<br>4,5-Di-O-methyl-8-prenylafzelechin-4beta-ol<br>3,5-Di-O-methyl-8-prenylafzelechin-4beta-ol<br>4,5-Di-O-methyl-8-prenylafzelechin-4beta-ol	3	Flavonoids	2.718
445.1870	0.78	M+Na-2H	C22H32O8	Didrovaltratum<br>4-(5'-((1E,3E,6Z,9Z)-dodeca-1,3,6,9-tetraen-1-yl)-[3,3'-bi(1,2-dioxolan)]-5-yl)-4-hydroperoxybutanoic acid<br>Dihydrovaltrate	3	Fatty acid	2.643

				8beta-Angeloyloxy-15-hydroxy-1alpha,10R-dimethoxy-3-oxo-11(13)-germacren-12,6alpha-olide			
448.1616	0.79	-	-	NA	4	-	2.793
387.1801	0.79	M+H	C22H26O6	3,5-Di-O-methyl-8-prenylafzelechin-4beta-ol<br>4,5-Di-O-methyl-8-prenylafzelechin-4beta-ol	3	Steroids	2.408
		M+Na	C20H28O6	Terpentecin<br>Gibberellin A44 diacid<br>Phorbol<br>3b-Hydroxy-6b-(2,3-epoxy-2-methylbutanoyloxy)-7(11)-eremophilen-12,8b-olide<br>12-Oxo-20-carboxy-leukotriene B4<br>3b,9b-Dihydroxy-6b-angeloyloxy-7(11)-eremophilen-12,8b-olide<br>Gibberellin A100<br>Gibberellin A74<br>3b,8a-Dihydroxy-6b-angeloyloxy-7(11)-eremophilen-12,8-olide<br>Gibberellin A97<br>Eremopetasitenin A1<br>Blumealactone A<br>Cinncassiol C2<br>Blumealactone B<br>3b,8b-Dihydroxy-6b-angeloyloxy-7(11)-eremophilen-12,8-olide<br>Gibberellin A123			
		M+H-H2O	C22H28O7	prednisolone-16alpha-carboxylic acid			
		2M+Na	C10H14O3	Iridotrial<br>5-oxo-7-decynoic acid<br>(-)-5-oxo-1,2-campholide<br>10-oxo-5,8-decadienoic acid<br>Furfuryl isovalerate<br>Furfuryl pentanoate<br>Iridotrial<br>(-)-5-oxo-1,2-campholide			
405.2102	0.79	M+H	C19H32O9	Pisumionoside	2	Terpene gycoside	2,422
388.1835	0.79		C20H27N3O6	Imidapril	3	Hipertension drug	2.425
106.0733	0.77	-	-	NA	4	-	2.686
432.2220	0.80	-	-	NA	4	-	2.393
795.3353	0.80	2M+Na	C22H26O6	3,5-Di-O-methyl-8-prenylafzelechin-4beta-ol<br>4,5-Di-O-methyl-8-prenylafzelechin-4beta-ol	3	Flavonoids	2.718
791.3832	0.80	2M+Na	C19H25BN4O4	Bortezomib	3	Drug	6.787
			C23H28O5	(6beta,7alpha,12beta,13beta)-7-Hydroxy-11,16-dioxo-8,14-apianadien-22,6-olide<br>Armillarivin			
520.3404	1.60	M+H	C26H50NO7P	PC(18:2(2E,4E)/0:0)<br>2-linoleoyl-sn-glycero-3-phosphocholine<br>PC(18:2(9Z,12Z)/0:0)[U]<br>PC(18:2(9Z,12Z)/0:0)	3	**Glycerophos phocholine**<br>Glicerophosp hoserine/ PC / PE/ Cardenolide glycoside	0.468
			C30H48O7	Cucurbitacin P<br>Hippurin-1<br>22-epi-Hippurin-1	3		
		M+Na	C24H52NO7P	1-(2-methoxy-octadecanyl)-sn-glycero-3-phosphoethanolamine	3		
			C35H46O2	Neurosporaxanthin	3		
		M+H-H2O	C26H52NO8P	PE(10:0/11:0)[U]<br>PE(11:0/10:0)[U]<br>PE(9:0/12:0)[U]<br>PC(16:0/2:0)[S]<br>PC(16:0/2:0)[U]<br>PC(9:0/9:0)[U]	2		

				PC(2:0/16:0)<br>PC(16:0/2:0)<br>PC(9:0/9:0)<br>PS(P-20:0/0:0)<br>**1-O-Palmitoyl-2-O-acetyl-sn-glycero-3-phosphorylcholine**			
		M+NH4	C29H44O7	Desglucocoroloside	3		
550.4241	7.10	M+H-H2O	C29H62NO7P	1-(2-methoxy-tricosanyl)-sn-glycero-3-phosphoethanolamine	3	Glicerophosphoethanolamine	0.489
536.4079	5.17	M+H	C35H53NO3	11alpha-(4-dimethylaminophenyl)-1alpha,25-dihydroxyvitamin D3 / 11alpha-(4-dimethylaminophenyl)-1alpha,25-dihydroxycholecalciferol	3	Phosphoethanolamine	0.464
			C28H58NO6P	PC(P-20:0/0:0)<br>PC(O-18:0/O-2:1(1E))			
		M+H-H2O	C28H60NO7P	1-(2-methoxy-docosanyl)-sn-glycero-3-phosphoethanolamine			
		2M+NH4	C12H25N3O3	Lysyl-Isoleucine<br>Lysyl-Leucine<br>Isoleucyl-Lysine<br>Leucyl-Lysine			
770.6063	11.09	M+H	C44H84NO7P	1-O-Hexadecyl-2-O-dihomogammalinolenoylglycero-3-phosphocholine<br>Different PCs	3	Phosphocholine, Phosphoethanolamine, glucosylceramide/ quinones	0.445
			C48H82O7	DG(11M5/13D5/0:0)<br>DG(13D5/11M5/0:0)<br>DG(13M5/11D5/0:0)<br>DG(11D5/13M5/0:0)			
		M+Na	C42H86NO7P	Different PEs and PCs	3		
			C53H80O2	Plastoquinone 9<br>Plastochromenol-8			
		M+H-H2O	C44H86NO8P	Different PEs, PE-NMes, PCs, DGs	3	Phosphocholines/ Diglycerides/ Phosphoethanolamine/Ceramides	
			C44H85NO10	GlcCer(t18:1(8Z)/20:0(2OH[S]))	3		
		M+NH4	C43H79NO9	Asperamide B<br>Flavicerebroside B<br>Flavuside B<br>GlcCer(d18:2(4E,8E)(9Me)/18:1(3E)(2OH[S]))<br>GalCer(d18:2(4E,8E)(9Me)/18:1(3E)(2OH[S]))	3		
494.3609	5.28	M+H	C25H52NO6P	PC(P-17:0/0:0)<br>PE(P-20:0/0:0)	3	PC/ PE/ Vitamins	0.421
		M+NH4	C32H44O3	(20S)-1alpha,25-dihydroxy-20-phenyl-16,17-didehydro-21-norvitamin D3 / (20S)-1alpha,25-dihydroxy-20-phenyl-16,17-didehydro-21-norcholecalciferol<br>(20R)-1alpha,25-dihydroxy-20-phenyl-16,17-didehydro-21-norvitamin D3 / (20R)-1alpha,25-dihydroxy-20-phenyl-16,17-didehydro-21-norcholecalciferol	3		
		M+H-H2O	C25H54NO7P	1-O-(2-methoxyhexadecyl)-sn-glycerol-3-phosphocholine<br>1-(2-methoxy-nonadecanyl)-sn-glycero-3-phosphoethanolamine	3		
508.3767	3.84	M+H	C26H54NO6P	PC(O-18:1(11Z)/0:0)<br>PC(O-18:1(9Z)/0:0)<br>PC(O-18:1(1E)/0:0)<br>PC(O-18:1(9E)/0:0)[S]<br>PC(O-18:1(9Z)/0:0)[S]<br>PC(O-18:1(9Z)/0:0)[U]<br>PC(P-18:0/0:0)	3	PC/ PE/ Xantines	0.445
		M+NH4	C33H46O3	7',8'-Dihydro-8'-hydroxyreticulataxanthin	3		

		M+H-H2O	C26H56NO7P	1-(2-methoxy-eicosanyl)-sn-glycero-3-phosphoethanolamine	3		
1484.140	9.18	2M+H	C42H80NO7P	Different PEs, PCs	3	PC/ PE	0.267
464.3147	2.29	-	-	NA	4	-	0.425

Table S3. Metabolites´ annotation from metabolome comparison of MCI-AD *vs.* healthy subjects.

Mass (*m/z*)	RT (min)	Adduct ion	Formula	Identification of variables		Compound class / Metabolism	FC[a]
				Metabolite annotation	Level #		
252.9830	1.27	-	-	NA	4	-	2.838
299.0264	1.03	2M-H	C4H6O6	D-Tartaric acid<br>2,3-Dihydroxybutanedioic acid	3	Food aditive<br>(tartaric acid)	2.144
362.2550	0.85	2M-H	C41H75O8P	Different Pas	3	Phosphatídic acid	0.340
			C41H74O10	1-O-beta-D-Glucopyranosyl-2,3-di-O-(8-hexadecenoyl)glycerol	3	Glycosyldiacylglycerol	
397.3438	5.05	-	-	NA	4	-	14.312
409.3113	6.92	M+Na-2H	C26H44O2	1alpha,25-dihydroxy-3-deoxy-19-norvitamin D3 / 1alpha,25-dihydroxy-3-deoxy-19-norcholecalciferol<br>1beta,25-dihydroxy-3-deoxy-19-norvitamin D3 / 1beta,25-dihydroxy-3-deoxy-19-norcholecalciferol<br>25-dihydroxy-19-nor-3-epivitamin D3 / 25-dihydroxy-19-nor-3-epicholecalciferol<br>25-dihydroxy-19-norvitamin D3 / 25-dihydroxy-19-norcholecalciferol<br>26:4(11Z,14Z,17Z,20Z)<br>7Z,13Z,16Z,19Z-Docosatetraenyl isobutyrate<br>3-Hydroxy-1-phenyl-1-eicosanone<br>C26:4	3	Vitamin<br>Fatty acyl<br>Long-chain fatty alcohol	0.136
425.3757	5.83	-	-	NA	4	-	15.231
518.3513	5.13	2M+NH4	C15H22O3	Ngaione (-)<br>Aspergillusene D<br>(+)-Armillarin<br>Pentalenic acid<br>1-deoxy-11beta-hydroxypentalenic acid<br>Mumiamicin<br>Ketosantalic acid<br>Procurcumadiol<br>Cadabicilone<br>3-Hydroxytrichothecene<br>(3beta,8beta)-3-Hydroxy-7(11)-eremophilen-12,8-olide<br>Abscisic alcohol<br>Valerenolic acid<br>Lactaronecatorin A<br>(6beta,8alpha)-6-Hydroxy-7(11)-eremophilen-12,8-olide<br>(3beta,6beta)-Furanoeremophilane-3,6-diol<br>Blennin A<br>Ketopelenolide a	3	Sesquiterpenoids<br>Furan fatty acid<br>Terpenoids<br>Sesquiterpene mycotoxins<br>Tepene lactones	3.313
741.5615	7.77	M+Na	C51H74O2	Dihydromenaquinone-8	3	Quinone	2.056
		M+NH4	C41H73NO9	bacteriohopane-31,32,33,34-tetrol-35-cyclitol	3		
766.5734	7.93	M+Na	C42H82NO7P	Different PEs, PCs, PnCs.	3	PEs/ PCs,/ PnCs/ glucosylceramide	2.342
			C42H81NO9	GlcCer(d14:1(4E)/22:0(2OH))<br>GlcCer(d16:1(4E)/20:0(2OH))<br>GlcCer(d18:1(8E)/18:0(2OH[R]))<br>GlcCer(d18:1(8Z)/18:0(2OH[R]))			
		M+H	C44H80NO7P	PC(P-16:0/20:4(8Z,11Z,14Z,17Z))<br>PC(P-18:0/18:4(6Z,9Z,12Z,15Z))	2	PC	

				PC(18:3(6Z,9Z,12Z)/P-18:1(9Z))<br>PC(P-18:1(11Z)/18:3(6Z,9Z,12Z))<br>PC(18:3(9Z,12Z,15Z)/P-18:1(9Z))<br>PC(18:3(6Z,9Z,12Z)/P-18:1(11Z))<br>PC(18:3(9Z,12Z,15Z)/P-18:1(11Z))<br>PC(18:4(6Z,9Z,12Z,15Z)/P-18:0)<br>PC(P-18:1(11Z)/18:3(9Z,12Z,15Z))<br>PC(P-18:1(9Z)/18:3(9Z,12Z,15Z))<br>PC(P-18:1(9Z)/18:3(6Z,9Z,12Z))<br>PC(20:4(5Z,8Z,11Z,14Z)/P-16:0)			
		M+H-H2O	C44H82NO8P	Different PCs, PE-NMe, Pes	3	PC/ PE/ PE-NMe	
767.5783	8.39	-	-	NA	4	-	2.063
876.3024	16.38	M+Na	C32H55NO25	Lacto-n-fucopentaose I<br>Lex-lactose<br>Lacto-N-fucopentaose V<br>Lacto-N-fucopentaose III<br>Lacto-N-fucopentaose-2	3	Fucopentaose	9.140
255.2429	1.40	-	-	NA	4	-	14.130
296.2946	5.00	M+NH4	C19H34O	6Z,9Z-Nonadecadien-3-one<br>9R,10S-Epoxy-3Z,6Z-nonadecadiene<br>9S,10R-Epoxy-3Z,6Z-nonadecadiene<br>6R,7S-Epoxy-3Z,9Z-nonadecadiene<br>6S,7R-Epoxy-3Z,9Z-nonadecadiene<br>9S,10R-Epoxy-6Z,12Z-nonadecadiene<br>3R,4S-Epoxy-6Z,9Z-nonadecadiene<br>3S,4R-Epoxy-6Z,9Z-nonadecadiene<br>trans-4S,5S-Epoxy-6Z,9Z-nonadecadiene<br>cis-6S,7R-Epoxy-9Z,12Z-nonadecadiene	3	Nonadecadiene	2.255
		M+H-H2O	C19H39NO2	Margaroyl-EA			
311.3054	3.68	-	-	NA	4	-	13.801
324.3259	5.84	M+NH4	C21H38O	3E,6Z-Heneicosadien-11-one<br>6Z,8E-Heneicosadien-11-one<br>6Z,9E-Heneicosadien-11-one<br>6Z,9Z-Heneicosadien-11-one<br>9R,10S-Epoxy-3Z,6Z-heneicosadiene<br>9S,10R-Epoxy-3Z,6Z-heneicosadiene<br>6R,7S-Epoxy-3Z,9Z-heneicosadiene<br>6S,7R-Epoxy-3Z,9Z-heneicosadiene<br>cis-3,4-Epoxy-6Z,9Z-heneicosadiene<br>trans-11S,12S-Epoxy-6Z,9Z-heneicosadiene	3	Heneicosadiene	2.356
339.3368	5.07	-	-	NA	4	-	15.231
350.3417	5.97	M+NH4	C23H40O	3-heptadecylphenol	3	Heptadecylphenol	2.831
352.3573	6.79	M+NH4	C23H42O	9,10-Epoxy-3Z,6Z-tricosadiene<br>6,7-Epoxy-3Z,9Z-tricosadiene	3	Tricosadiene	2.084
		M+H-H2O	C23H47NO2	Penazetidine A	3	Azetidine	
367.3682	5.85	-	-	NA	4	-	14.753
